# Epstein-Barr virus Latent Membrane Protein LMP1 reduces p53 protein levels independent of the PI3K-Akt pathway

**DOI:** 10.1186/1756-0500-4-551

**Published:** 2011-12-21

**Authors:** Roslina Husaini, Munirah Ahmad, Alan Soo-Beng Khoo

**Affiliations:** 1 Molecular Pathology Unit, Cancer Research Centre, Institute for Medical Research, Jalan Pahang, 50588 Kuala Lumpur, Malaysia

**Keywords:** Nasopharyngeal carcinoma (NPC), Epstein-Barr virus, Latent membrane protein, p53 tumour suppressor gene, ubiquitination

## Abstract

**Background:**

Nasopharyngeal carcinoma (NPC) is an epithelial malignancy, which commonly occurs in Southern China, Taiwan, North Africa and Southeast Asia. Nasopharyngeal carcinoma is strongly associated with Epstein-Barr virus infection. The p53 tumour suppressor protein is rarely mutated in NPC suggesting that the inactivation of p53 pathway in NPC could be due to the presence of EBV proteins. The aim of this work was to determine the effects of EBV proteins namely LMP1 and LMP2A on the expression levels of p53 protein.

**Findings:**

In this work we found that LMP1, but not LMP2A, decreased p53 protein levels. Overexpression of LMP1 resulted in increased ubiquitination of p53 suggesting that the decreased p53 protein levels by LMP1 was due to increased degradation of the protein. The reduction of p53 protein levels was independent of the PI3K-Akt pathway.

**Conclusions:**

LMP1, but not LMP2A, reduced p53 protein levels through the increase in the polyubiquitination of p53 protein and was independent of the PI3K-Akt pathway.

## Background

Nasopharyngeal carcinoma (NPC) is an epithelial malignancy which mostly occurs in Southern China, Hong Kong, Taiwan, North Africa and Southeast Asia [1] including Malaysia [2,3]. In contrast to other head and neck cancers and other epithelial malignancies, NPC is often associated with Epstein-Barr virus (EBV) infection, a ubiquitous infectious agent, suggesting that NPC tumourigenesis involves EBV infection [4].

Epstein-Barr virus (EBV) is a human gamma herpes virus which primarily involves infection of B lymphocytes and certain epithelial cells. It was found to efficiently cause transformation of human primary B lymphocytes, both *in vitro and in vivo*, into immortalized lymphoblastoid cell lines (LCL) that proliferate indefinitely by harbouring the virus in its latent state [5]. The linear double-stranded EBV DNA genome is approximately 172 kb in length which encodes about 85 genes. These genes include three latent membrane proteins 1, 2A, and 2B (LMPs), six EBV nuclear antigens (EBNA1, 2, 3A, 3B, 3C, and EBNA-LP), and two small, non-coding nuclear RNAs (EBERs). Of these, LMP1 is oncogenic and was reported to be expressed in more than 70% of NPC patients [6,7].

LMP1 is a transmembrane protein and is essential for transformation. LMP1 is able to induce tumour invasiveness and metastasis. A study by Wakisaka & Pagano has shown that LMP1 induces matrix metalloproteinase 9 (MMP-9), an enzyme that disrupts the basement membrane [8]. Gene transfer studies showed that LMP1 represses apoptosis in B cells by upregulation of anti-apoptotic protein, Bcl-2 [9-11]. Another EBV transmembrane protein, LMP2A, has been shown to inhibit an epithelial cell line, HaCat, from differentiating in organotypic raft cultures [12].

The p53 tumour suppressor protein is a sequence-specific DNA damage-inducible transcription factor that controls cell growth by regulating cell apoptosis and G1 cell cycle arrest, mainly by upregulating Bax and the cyclin-dependent kinase inhibitor p21/WAF1/CIP1, respectively [13]. p53 is activated upon exposure to genotoxic stress, which then upregulates the expression of p21 resulting in a halt in cell cycle progression to allow repair of damaged DNA. However, if the damage is too severe, p53 then induces apoptosis [14,15]. A few reports have suggested that EBV may interfere with cell cycle checkpoints at both G_1_/S and G_2_/M [16,17]. EBV might also target p53 upstream pathways such as Chk2 and may affect p53 stability [16,18]. LMP1 was also shown to interfere with the growth suppression induced by wild-type p53.

Several studies have shown that the p53 protein was overexpressed in many cases of NPC as detected by immunochemistry [19-21]. This is further supported by the work in our laboratory which showed that p53 protein was found to be accumulated in 58 NPC biopsy samples formalin-fixed, paraffin-waxed embedded tissues of Malaysian patients but the p53 gene was not mutated at the p53 mutation hot-spots Exons 5-8 [22]. Liu and co-workers found that LMP1 repressed p53 from mediating DNA repair and inactivated p53 transcriptional activity [23]. This however contradicts with the findings by Li and co-workers who found that LMP1 activated p53 transcriptional activity and increased its stability through multi-sites phosphorylation of p53 protein resulting in the accumulation of p53 protein in the nucleus [24,25].

We sought to verify the effect of LMP1 on p53 protein levels in a heterologous system, the U2OS osteosarcoma cell line, which is known to harbour wild type p53 and is a commonly used model system to study the p53 pathway.

## Methods

### Cell Cultures

U2OS, a human osteosarcoma cell line, was maintained in Dulbecco's modified Eagle's medium (DMEM) supplemented with 10% (v/v) foetal calf serum (FCS), 100 U/ml penicillin and 100 μg/ml streptomycin (Invitrogen, Auckland, New Zealand) in a humidified atmosphere containing 5% (v/v) CO_2 _at 37°C. EBV-negative NPC cells (HONE1), EBV infected NPC cells (HONE-Akata), stable cell lines CNE1-pBabe and CNE1-pBabe LMP1 were all grown under similar conditions.

### Plasmids

Plasmids used in this study were empty vector plasmids pcDNA3.1(-), pcDNA3.1(+), and pSG5 and plasmid with LMP1 insert pcDNA3.1(+)LMP1 (a kind gift from Cesarman E, Cornell University, USA), and plasmids with LMP2A-HA insert pcDNA3.1(-)LMP2A-HA and pSG5.LMP2A-HA (a kind gift from R. Longnecker, Northwestern University, USA) and pcDNA3.1(+)EBNA1 (a kind gift from Christian Munz, Rockefeller University, USA). Hup53 gene was subcloned from pT7.7Hup53 construct (bacterial expression plasmid) (a kind gift of Steven Picksley, University of Bradford, UK) into the pcDNA3.1 mammalian expression vector to generate pcDNA3.1(-)Hup53. We also employed dominant negative Akt construct pcDNA3 DN Akt plasmid (a kind gift of P.P. Pandolfi, Memorial Sloan Kettering Cancer Centre, USA). pEGFP plasmid was co-transfected to determine transfection efficiency.

### Antibodies

Primary antibodies used in this work were anti-p53 mouse monoclonal antibody (DO-1) (Santa Cruz, California, USA) at 1:1000 dilution, anti-LMP1 mouse monoclonal antibody (DAKO, Denmark) at 1:500 dilution, rat monoclonal EBV LMP2A, Clone 14B7 (E. Kremmer, Institute for Molecular Immunology, Munich) at 1:50 dilution, anti-Akt rabbit polyclonal antibody (Cell Signalling Technology, Danvers, USA) at 1:1000 dilution, anti-ubiquitin rabbit polyclonal antibody (Sigma, Saint Louis, USA) at 1:100 dilution, and anti-β-actin mouse monoclonal antibody (Santa Cruz, California, USA) at 1:1000 which served as a loading control protein. Secondary antibodies used were rabbit anti-mouse polyclonal antibody (DAKO, Denmark) at 1:2000 dilution, anti-rat Fab HRP/ECL at 1:10000 dilution and anti-rabbit polyclonal antibody (Cell Signalling Technology, Danvers, USA) at 1:15000 dilution.

### Western blotting

**Figure 1:**
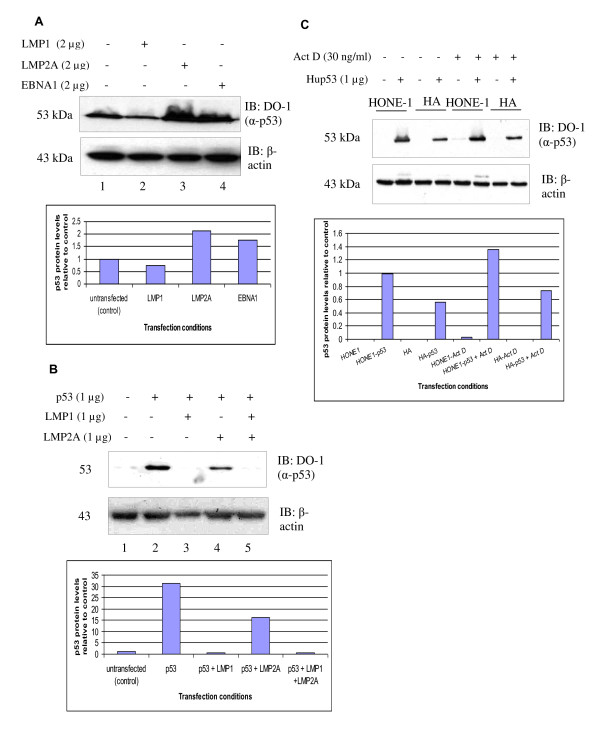
**LMP1, but not LMP2A or EBNA1, reduces p53 protein levels**. **(A) **Western blot analysis of the effects of LMP1, LMP2A and EBNA1 on the expression levels of endogenous p53 protein in U2OS cells. Transfection of LMP1 reduced the level of p53 protein. **(B) **Co-transfection of LMP1 abolished p53 protein level in HONE1 NPC cells transiently transfected with p53. A slight reduction in the exogenous p53 protein level in LMP2A co-transfected in HONE1 NPC cells setting but not in U2OS cells in (A). (**C**) EBV-negative HONE1 and EBV-positive HONE Akata (HA) cells were transfected with p53 construct. p53 protein levels were determined in the Actinomycin-D induced and uninduced state. EBV-positive HA NPC cells have lower levels of p53 protein in comparison with EBV-negative HONE NPC cells. β-actin was served as a loading control in all the three experiments. Representative blots were quantitated using Image J.

**Figure 2:**
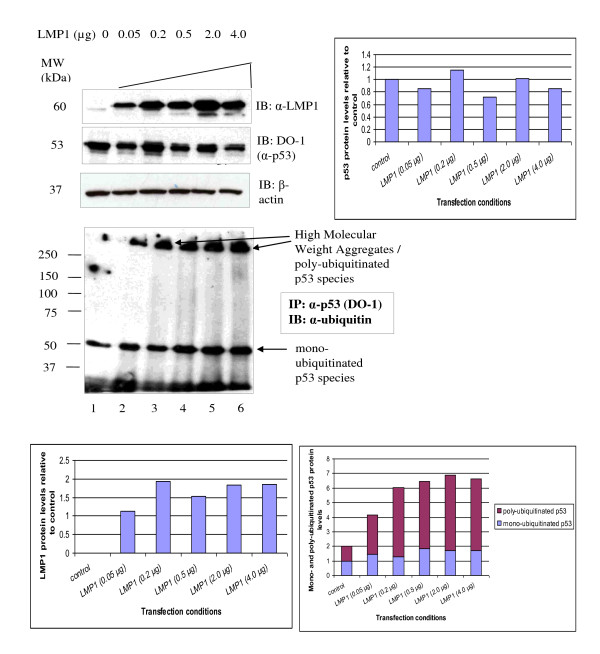
**Polyubiquitinated p53 species detected in U2OS cells titrated with LMP1**. Western blot detection of transfected LMP1, endogenous p53 protein levels and detection of ubiquitinated p53 species in U2OS cells transiently transfected with titrated LMP1 (0.05 - 4.0 μg). For the ubiquitination experiment, p53 protein was immunoprecipitated from the cell lysate with anti-p53 mouse monoclonal antibody (DO-1) and then immunoblotted with anti-ubiquitin rabbit polyclonal antibody. β-actin was served as a loading control in the experiment. Representative blots were quantitated using Image J. Increased polyubiquitinated p53 protein was seen as high molecular weight aggregates in LMP1 expressing cells.

**Figure 3:**
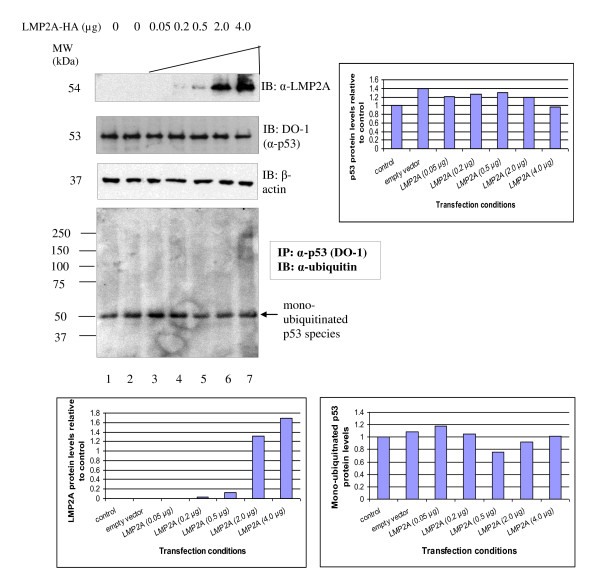
**Only monoubiquitinated p53 species detected in U2OS titrated with LMP2A**. Western blot detection of transfected LMP2A-HA, endogenous p53 protein levels and ubiquitinated p53 species in U2OS cells transiently transfected with titrated LMP2A-HA (0.05 - 4.0 μg). For the ubiquitination experiment, p53 protein was immunoprecipitated from the cell lysate with anti-p53 mouse monoclonal antibody (DO-1) and then immunoblotted with anti-ubiquitin rabbit polyclonal antibody. β-actin was served as a loading control in the experiment. Representative blots were quantitated using Image J. No polyubiquitinated p53 protein was seen in LMP2A expressing cells.

**Figure 4:**
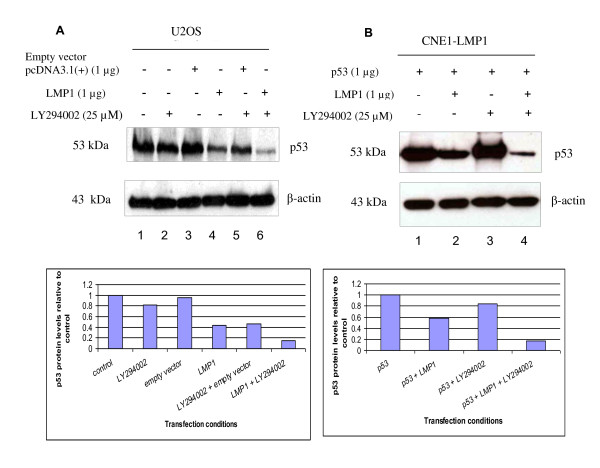
**PI3K-Akt inhibitor LY294002 does not restore p53 protein levels**. **(A) **Western blot analysis of the effects of PI3K inhibitor LY294002 on the endogenous p53 protein levels in U2OS cells. The cells were treated overnight, with 25 μM LY294002 or vehicle alone (DMSO). Treatment by LY294002 did not rescue the reduction of p53 protein levels by LMP1. **(B) **Western blot analysis of the effects of PI3K inhibitor LY294002 on the transfected p53 protein levels in CNE1-LMP1 stable cell line. The cells were treated overnight with 25 μM LY294002 or vehicle alone (DMSO). β-actin was served as a loading control in both the experiments. Representative blots were quantitated using Image J. Treatment with LY294002 did not rescue the reduction of p53 protein levels by LMP1.

Plasmid DNA was transfected into U2OS cells using Lipofectamine 2000 (Invitrogen, Auckland, New Zealand) according to the manufacturer's instructions. After 6 h of post-transfection, the cells were either left untreated (as control) or treated with 30 ng/ml Actinomycin D (Act D) (Sigma, Saint Louis, USA) or 25 μM LY294002 (Cell Signaling Technology, Danvers, USA) and incubated for 24 h before being harvested and subjected to Western blotting. After transfection and treatment with Actinomycin-D (Act D) (Sigma, Saint Louis, USA) or PI3K inhibitor, LY294002 (Cell Signalling Technology, Danvers, USA), or vehicle alone (as control), the cells were harvested and lysed using RIPA lysis buffer (1% NP-40, 1% sodium deoxycholate, 0.1% SDS, 150 mM NaCl, 10 mM Na_2_HPO_4 _pH 7.2) supplemented with protease inhibitor cocktail (Roche, Mannheim, Germany), 0.2 mM PMSF and 2 mM DTT. Total protein from the cell lysates was quantitated using Bradford Protein Assay (BioRad, California, USA). Equal amount of total protein (20 μg) was loaded onto 10-15% SDS-PAGE gels and eletroblotted onto polyvinyl difluoride (PVDF) membranes (Millipore, Bedford, USA) at 15 V overnight at room temperature. Following this, the membranes were then blocked with 5% non-fat dry milk in TBST (30 mM Tris HCl pH7.3, 0.1 M NaCl, 0.1% Tween 20) and incubated with primary antibodies and secondary antibodies for 1 hour each at room temperature. The proteins were visualised with Western LightningTM Chemiluminescence Reagent Plus (Perkin Elmer, Boston, USA), AmershamTM ECLTM Western Blotting Detection Reagents (GE Healthcare, Buckinghamshire, UK), or Super Signal^R ^West Femto (Pierce, Roskford, USA). Representative results of at least 2 independent experiments were shown in Figures 1, 2, 3, 4, &5. The signals were quantitated using Image J (NIH, Bethesda, USA).

**Figure 5:**
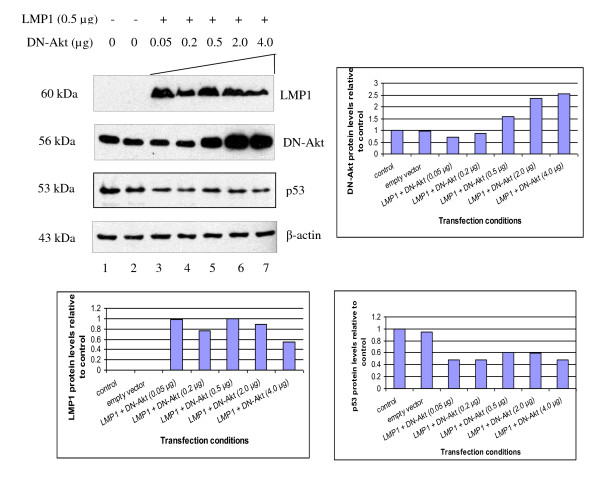
**Dominant-negative Akt (DN-Akt) does not restore p53 protein levels**. Western blot detection of LMP1, Dominant-negative Akt (DN-Akt) and p53 protein levels in U2OS cells transfected with fixed amount of LMP1 (0.5 μg) and titrated with DN-Akt (0.05 - 4.0 μg). β-actin was served as a loading control in the experiment. Representative blots were quantitated using Image J. Increasing amounts of DN-Akt (detected by anti-Akt antibodies) did not rescue reduction of p53 protein levels by LMP1.

### Immunoprecipitation (IP)

The cell lysates were first pre-cleared by incubating 100 μg total protein from each sample with 50% Protein G slurry (Upstate Cell Signaling, California, USA) on ice for 30 min with occasional mixing. After centrifugation, the supernatants were incubated with 1 μg anti-p53 monoclonal antibody (DO-1) (Santa Cruz, California, USA) overnight at 4°C on a rotator. Protein G slurry was added to each sample and rotated for 1 hr at 4°C. The p53 protein from the immuno-complexes were then eluted with SDS sample buffer, separated by SDS-PAGE, and immunoblotted with anti-ubiquitin rabbit polyclonal antibody (1:100 dilution) (Sigma, Saint Louis, USA) as the primary antibody and the secondary antibody was anti-rabbit polyclonal antibody (1:15000 dilution) (Cell Signaling Technology, Danvers, USA). Representative results of at least 2 independent experiments were shown in Figures 2 and 3. The signals were quantitated using Image J (NIH, Bethesda, USA).

(Our data is not yet publically available.)

## Results

### 1. Effects of LMP1 or LMP2A or both on p53 protein levels in U2OS cells

We tested the effects of individual EBV proteins LMP1, LMP2A-HA and EBNA1 on endogenous p53 levels in U2OS cells by transient transfection of these genes into the cells. Out of the three EBV proteins, only LMP1 clearly reduced the levels of endogenous p53 protein in U2OS cells (Figure 1(a), Lane 2). The p53 protein levels in cells transfected with LMP2A-HA and EBNA1 (Figure 1(a), Lanes 3 and 4) did not seem to be reduced in comparison to its basal levels in the control cells (Figure 1(a), Lane 1).

### 2. Effect of LMP1 or LMP2A or both on p53 protein levels in NPC cells

Because p53 was barely detectable in NPC cell lines, we transiently transfected the NPC cells with p53 construct in order to study the effects of EBV proteins on exogenous p53 protein levels. We used EBV-negative HONE1 NPC cells to study the effects of EBV LMP1 alone or EBV LMP2A alone or both on the levels of overexpressed p53 proteins. p53 protein level in HONE1 transfected with empty vector pcDNA3.1(+) was barely detectable (Figure 1(b), Lane 1). p53 protein was highly expressed in HONE1 transfected with p53 construct (Figure 1(b), Lane 2). HONE1 cells co-transfected with LMP1 and p53 showed absence of p53 protein or, in other words, LMP1 abolished p53 protein (Figure 1(b), Lane 3). However, LMP2A-HA only slightly reduced the level of p53 protein in HONE1 cells transfected with both LMP2A-HA and p53 (Figure 1(b), Lane 4). Co-transfection of p53, LMP1 and LMP2A-HA again resulted in the loss of p53 protein (Figure 1(b), Lane 5).

We transiently transfected EBV negative HONE1 and EBV positive HONE-Akata cells with p53 construct (Figure 1(c)). Untransfected cells were used as negative control. The cells after 6 hr post-transfection were then either treated with 30 ng/ml Actinomycin-D (Act-D) for 18 hr or left untreated. As determined in our earlier experiment, p53 protein was barely detectable in NPC cell lines and only slightly induced by Act-D (data not shown). No p53 protein was detected in untransfected cells. Upon treatment with Act-D, the p53 protein was hardly induced. EBV-positive HONE-Akata NPC cells had lower levels of p53 protein in comparison with EBV-negative HONE1 both in Act-D induced and uninduced state.

### 3. Immunoprecipitation study on ubiquitinated p53 species in U2OS cells titrated with either LMP1 or LMP2A

p53 protein levels in normal cells are kept in check by MDM2 protein which acts as a ubiquitin ligase that promotes p53 protein degradation by proteosome. The degradation of p53 protein then results in the low expression levels of MDM2 protein, which is a p53 transcriptional target gene, resulting in an autoregulatory feedback loop between p53 and MDM2 proteins [26-29].

We hypothesised that the mechanism of action for the reduction of p53 protein levels by LMP1 is through ubiquitination of p53 protein. In order to test this hypothesis, we immunoprecipitated p53 protein with anti-p53 mouse monoclonal antibody (DO-1) in U2OS titrated with increasing amounts of LMP1 and carried out Western blot with anti-ubiquitin rabbit polyclonal antibody. Direct Western blot (Figure 2) confirmed that LMP1 protein levels increased when LMP1 was increased (Lanes 2-6). Increasing in amounts of LMP1 resulted in decreasing levels of the p53 protein (Lanes 3-6). Monoubiquitinated p53 species slightly increased in levels. Increasing levels of LMP1 resulted in increased levels of the polyubiquitinated p53 species (Figure 2, Lanes 1-6).

In contrast, LMP2A-HA did not reduce p53 protein levels in U2OS cells (Figure 3, Lanes 3-7). The p53 protein levels in LMP2A-HA transfected cells (Figure 3, Lanes 3-7) were similar to the basal p53 protein level in untransfected U2OS cells and in U2OS cells transfected with empty vector control (Figure 3, Lanes 1 and 2, respectively). Similar levels of monoubiquitinated p53 species were observed in all samples irrespective of transfection (Figure 3, Lanes 1-7). However, no polyubiquitinated p53 species were seen in LMP2A-HA transfected U2OS cells (Figure 3, Lanes 3-7) compared to untransfected control (Figure 3, Lane 1).

### 4. Reduction of p53 protein levels by LMP1 is not dependent on the PI3K-Akt Pathway

LMP1 is known to activate the PI3Kinase/Akt pathway. Akt activation has been shown to phosphorylate MDM2 and induces its migration into the nucleus where it binds, ubiquitinates and degrades p53 protein resulting in a lower level of p53 protein [30]. Therefore we hypothesised that LMP1 reduces the p53 protein levels through the PI3K/Akt pathway.

To determine whether LMP1 reduces p53 protein levels through activation of the PI3K/Akt pathway in U2OS cells, we overexpressed LMP1 in the cells in the presence or absence of PI3K inhibitor, LY294002. As expected, the p53 level was lower in U2OS cells overexpressing LMP1 compared with the control (Figure 4(a), Lanes 4 and 1, respectively). Treatment with LY294002 did not affect the reduction of p53 levels by LMP1 (Figure 4(a), Lane 6). Since we have established NPC cells stably expressing LMP1, we then transfected CNE1-pBabe and CNE1-LMP1 NPC stable cells with p53 construct, at 6 hr post-transfection, treated with either 25 μM LY294002 overnight (18 hr) or vehicle alone (DMSO). In the presence of LMP1 in CNE1-LMP1 cells, the levels of transfected p53 protein were greatly reduced (Figure 4(b), Lanes 2 and 4) when compared to its basal levels in CNE1 with empty vector CNE1-pBabe cells (Figure 4(b), Lanes 1 and 3) regardless of the presence or absence of LY294002.

In order to further verify the results, we transfected U2OS cells with increasing amount of dominant negative Akt (DN-Akt) to inhibit the PI3K/Akt pathway in the presence of a fixed amount of LMP1. Expression of LMP1 resulted in a reduction in the basal p53 protein level. Increasing amount of DN-Akt did not rescue the reduction of p53 protein levels by LMP1 (Figure 5). In conclusion, the PI3K/Akt pathway was not involved in the reduction of p53 protein levels by LMP1 in U2OS cells.

## Discussion

The aim of this work was to determine the effects of EBV LMP1 and LMP2A on the levels of p53 protein. In order to do this, we transfected U2OS cells with either LMP1, LMP2A or EBNA1. LMP1 obviously reduced the levels of p53 protein when compared to its basal level in U2OS transfected with empty vector control. The levels of p53 protein did not seem to be reduced in U2OS cells transfected with LMP2A-HA and EBNA1 in comparison to its basal levels in the control cells.

We then verified the results by transiently transfecting HONE1 NPC cells with p53 construct, either with or without LMP1, or LMP2A, or both. In this study we found that LMP1 reduced the expression of p53 protein. We then proceeded to check the expression levels of endogenous and exogenous p53 protein levels in EBV negative HONE1 and EBV positive HONE-Akata cells, either treated or untreated with Actinomycin D. We found that EBV-positive HONE-Akata NPC cells had lower levels of p53 protein in comparison to EBV-negative HONE1 NPC cells.

In this work we have shown that LMP1 reduced or totally abolished the exogenous p53 protein levels in HONE1 cells co-transfected with p53 and LMP1 and also reduced endogenous p53 protein levels in U2OS cells transfected with LMP1. However, this is in contrast to Li L. and colleagues (2008) who showed that LMP1 induced accumulations of p53 protein [25]. In order to determine the mechanism of reduction of p53 protein levels by LMP1, we treated U2OS cells either with DMSO (vehicle control) or PI3K inhibitor LY294002, where the cells were either transfected or untransfected with LMP1 construct. p53 protein levels were reduced in the presence of LMP1 and were not rescued when the cells were treated with LY294002. In CNE1-LMP1 stable cells transfected with p53 construct and then treated with LY294002, the p53 protein levels were not rescued in comparison with its counterpart CNE1-pBabe stable cells transfected with p53 construct in the absence of LMP1. We then examined the effects of titrated Dominant negative Akt (DN-Akt) on the levels of p53 protein in the absence or presence of LMP1 in U2OS cells. We found that increasing in the amounts of DN-Akt did not restore the p53 protein levels to its basal levels both in untransfected U2OS cells and U2OS cells transfected with empty vector control. Taken together these results indicate that the reduction of p53 protein levels by LMP1 does not involve the PI3K-Akt pathway.

As p53 protein is ubiquitinated to target it for degradation, we then asked if overexpression of the latent membrane proteins affects the levels of ubiquitinated p53. We found that increasing the dosage of LMP1 resulted in a gradual reduction of endogenous p53 protein levels whereas titration of LMP2A-HA showed no reduction of endogenous p53 protein levels as compared to its basal level. Interestingly, mono- and poly-ubiquitinated p53 species were found in U2OS titrated with LMP1 whereas only mono-ubiquitinated p53 species were observed in U2OS cells irrespective of amount of LMP2A-HA transfected. This is the first time in which it was demonstrated that U2OS cells transfected with titrated LMP1 showed a distinct pattern of ubiquitination from U2OS cells transfected with titrated LMP2A-HA. This is in concordance with the findings that mono-ubiquitination acts as a signal for p53 nuclear export whereas poly-ubiquitinated p53 species are targeted for 26S proteosome degradation [31].

## Conclusions

We found that LMP1, but not LMP2A, decreased p53 protein levels. Overexpression of LMP1 resulted in increased polyubiquitination of p53 suggesting that the decreased p53 protein levels by LMP1 was due to increased degradation of the protein. The reduction of p53 protein levels was independent of the PI3K-Akt pathway.

## Competing interests

The authors declare that they have no competing interests.

## Authors' contributions

RH carried out the experiments, data analysis and drafted the manuscript. MA was involved in the experimental design and co-supervised the work. ASBK directed the study, reviewed the results, revised the draft manuscript and was the principal investigator for the study. All authors read and approved the final manuscript.

## References

[B1] Ferlay J GLOBOCAN 2000: Cancer incidence, mortality and prevalence worldwide (version 1.0) 2001 IARC Press, Lyon Version 1.0

[B2] Prasad U Pua KC Nasopharyngeal carcinoma: a delay in diagnosis The Medical journal of Malaysia 2000 55 230 235 19839151

[B3] Pua KC Khoo AS Yap YY Subramaniam SK Ong CA Gopala Krishnan G Shahid H Nasopharyngeal Carcinoma Database The Medical journal of Malaysia 2008 63 Suppl C 59 62 19230249

[B4] Pathmanathan R Prasad U Sadler R Flynn K Raab-Traub N Clonal proliferations of cells infected with Epstein-Barr virus in preinvasive lesions related to nasopharyngeal carcinoma The New England journal of medicine 1995 333 693 698 10.1056/NEJM199509143331103 7637746

[B5] Kieff E Epstein-Barr virus--increasing evidence of a link to carcinoma The New England journal of medicine 1995 333 724 726 10.1056/NEJM199509143331110 7637753

[B6] Vera-Sempere FJ Burgos JS Botella MS Cordoba J Gobernado M Immunohistochemical expression of Epstein-Barr virus-encoded latent membrane protein (LMP-1) in paraffin sections of EBV-associated nasopharyngeal carcinoma in Spanish patients Eur J Cancer B Oral Oncol 1996 32B 163 168 8762873 10.1016/0964-1955(95)00093-3

[B7] Lin SY Tsang NM Kao SC Hsieh YL Chen YP Tsai CS Kuo TT Hao SP Chen IH Hong JH Presence of Epstein-Barr virus latent membrane protein 1 gene in the nasopharyngeal swabs from patients with nasopharyngeal carcinoma Head Neck 2001 23 194 200 10.1002/1097-0347(200103)23:3<194::AID-HED1018>3.0.CO;2-X 11428449

[B8] Wakisaka N Pagano JS Epstein-Barr virus induces invasion and metastasis factors Anticancer Res 2003 23 2133 2138 12894587

[B9] Gregory CD Dive C Henderson S Smith CA Williams GT Gordon J Rickinson AB Activation of Epstein-Barr virus latent genes protects human B cells from death by apoptosis Nature 1991 349 612 614 10.1038/349612a0 1705663

[B10] Henderson S Rowe M Gregory C Croom-Carter D Wang F Longnecker R Kieff E Rickinson A Induction of bcl-2 expression by Epstein-Barr virus latent membrane protein 1 protects infected B cells from programmed cell death Cell 1991 65 1107 1115 10.1016/0092-8674(91)90007-L 1648447

[B11] Laherty CD Hu HM Opipari AW Wang F Dixit VM The Epstein-Barr virus LMP1 gene product induces A20 zinc finger protein expression by activating nuclear factor kappa B The Journal of biological chemistry 1992 267 24157 24160 1332946

[B12] Raab-Traub N Epstein-Barr virus in the pathogenesis of NPC Semin Cancer Biol 2002 12 431 441 10.1016/S1044579X0200086X 12450729

[B13] el-Deiry WS Tokino T Velculescu VE Levy DB Parsons R Trent JM Lin D Mercer WE Kinzler KW Vogelstein B WAF1, a potential mediator of p53 tumor suppression Cell 1993 75 817 825 10.1016/0092-8674(93)90500-P 8242752

[B14] Kastan MB Onyekwere O Sidransky D Vogelstein B Craig RW Participation of p53 protein in the cellular response to DNA damage Cancer research 1991 51 6304 6311 1933891

[B15] Yonish-Rouach E Resnitzky D Lotem J Sachs L Kimchi A Oren M Wild-type p53 induces apoptosis of myeloid leukaemic cells that is inhibited by interleukin-6 Nature 1991 352 345 347 10.1038/352345a0 1852210

[B16] Krauer KG Burgess A Buck M Flanagan J Sculley TB Gabrielli B The EBNA-3 gene family proteins disrupt the G2/M checkpoint Oncogene 2004 23 1342 1353 10.1038/sj.onc.1207253 14716295

[B17] Wade M Allday MJ Epstein-Barr virus suppresses a G(2)/M checkpoint activated by genotoxins Mol Cell Biol 2000 20 1344 1360 10.1128/MCB.20.4.1344-1360.2000 10648620 PMC85280

[B18] Saridakis V Sheng Y Sarkari F Holowaty MN Shire K Nguyen T Zhang RG Liao J Lee W Edwards AM Structure of the p53 binding domain of HAUSP/USP7 bound to Epstein-Barr nuclear antigen 1 implications for EBV-mediated immortalization Mol Cell 2005 18 25 36 10.1016/j.molcel.2005.02.029 15808506

[B19] Taweevisit M Overexpression of p53 and neoplastic cell proliferation in undifferentiated nasopharyngeal carcinoma Southeast Asian J Trop Med Public Health 2007 38 136 140 17539259

[B20] Gulley ML Burton MP Allred DC Nicholls JM Amin MB Ro JY Schneider BG Epstein-Barr virus infection is associated with p53 accumulation in nasopharyngeal carcinoma Human pathology 1998 29 252 259 10.1016/S0046-8177(98)90044-2 9496828

[B21] Porter MJ Field JK Lee JC Leung SF Lo D Van Hasselt CA Detection of the tumour suppressor gene p53 in nasopharyngeal carcinoma in Hong Kong Chinese Anticancer Res 1994 14 1357 1360 8067705

[B22] Hoe SL Lee ES Khoo ASB Peh SC p53 and nasopharyngeal carcinoma: a Malaysian study Pathology 2009 41 561 565 10.1080/00313020903071504 19900105

[B23] Liu MT Chang YT Chen SC Chuang YC Chen YR Lin CS Chen JY Epstein-Barr virus latent membrane protein 1 represses p53-mediated DNA repair and transcriptional activity Oncogene 2005 24 2635 2646 10.1038/sj.onc.1208319 15829976

[B24] Li L Guo L Tao Y Zhou S Wang Z Luo W Hu D Li Z Xiao L Tang M Latent membrane protein 1 of Epstein-Barr virus regulates p53 phosphorylation through MAP kinases Cancer letters 2007 255 219 231 10.1016/j.canlet.2007.04.014 17582679

[B25] Li L Zhou S Chen X Guo L Li Z Hu D Luo X Ma X Tang M Yi W The activation of p53 mediated by Epstein-Barr virus latent membrane protein 1 in SV40 large T-antigen transformed cells FEBS Lett 2008 582 755 762 10.1016/j.febslet.2008.01.031 18242176

[B26] Haupt Y Maya R Kazaz A Oren M Mdm2 promotes the rapid degradation of p53 Nature 1997 387 296 299 10.1038/387296a0 9153395

[B27] Kubbutat MH Jones SN Vousden KH Regulation of p53 stability by Mdm2 Nature 1997 387 299 303 10.1038/387299a0 9153396

[B28] Midgley CA Lane DP p53 protein stability in tumour cells is not determined by mutation but is dependent on Mdm2 binding Oncogene 1997 15 1179 1189 10.1038/sj.onc.1201459 9294611

[B29] Fuchs SY Adler V Buschmann T Wu X Ronai Z Mdm2 association with p53 targets its ubiquitination Oncogene 1998 17 2543 2547 10.1038/sj.onc.1202200 9824166

[B30] Ogawara Y Kishishita S Obata T Isazawa Y Suzuki T Tanaka K Masuyama N Gotoh Y Akt enhances Mdm2-mediated ubiquitination and degradation of p53 The Journal of biological chemistry 2002 277 21843 21850 10.1074/jbc.M109745200 11923280

[B31] Li M Brooks CL Wu-Baer F Chen D Baer R Gu W Mono- versus polyubiquitination: differential control of p53 fate by Mdm2 Science 2003 302 New York, NY 1972 1975 10.1126/science.1091362 14671306

